# High temporal-resolution scanning transmission electron microscopy using sparse-serpentine scan pathways

**DOI:** 10.1038/s41598-021-02052-1

**Published:** 2021-11-22

**Authors:** Eduardo Ortega, Daniel Nicholls, Nigel D. Browning, Niels de Jonge

**Affiliations:** 1 INM – Leibniz Institute for New Materials, 66123 Saarbrucken, Germany; 2 School of Engineering & School of Physical Sciences, University of Liverpool, Liverpool, L69 3GQ UK; 3 Sivananthan Laboratories, 590 Territorial Drive, Bolingbrook, IL 60440 USA; 4 Department of Physics, Saarland University, 66123 Saarbrucken, Germany

**Keywords:** Scanning electron microscopy, Transmission electron microscopy, Nanoparticles

## Abstract

Scanning transmission electron microscopy (STEM) provides structural analysis with sub-angstrom resolution. But the pixel-by-pixel scanning process is a limiting factor in acquiring high-speed data. Different strategies have been implemented to increase scanning speeds while at the same time minimizing beam damage via optimizing the scanning strategy. Here, we achieve the highest possible scanning speed by eliminating the image acquisition dead time induced by the beam flyback time combined with reducing the amount of scanning pixels via sparse imaging. A calibration procedure was developed to compensate for the hysteresis of the magnetic scan coils. A combination of sparse and serpentine scanning routines was tested for a crystalline thin film, gold nanoparticles, and in an in-situ liquid phase STEM experiment. Frame rates of 92, 23 and 5.8 s^-1^ were achieved for images of a width of 128, 256, and 512 pixels, respectively. The methods described here can be applied to single-particle tracking and analysis of radiation sensitive materials.

## Introduction

Scanning transmission electron microscopy (STEM) has become the technique of choice for imaging, structure, and spectroscopy analysis with atomic level resolution^1,2^. The implementation of spherical aberration correctors (Cs-Corr STEM) has allowed for sub-angstrom resolution^3^; while the introduction of pixelated detectors has enabled the recovery of electron-phase information to even perform electric- and magnetic field measurements^4,5^. These technological advances have been accompanied by an increased beam current density, caused by the reduced electron probe, and increased dwell times *T*_*D*_, needed to record entire diffraction patterns at each scan position within a 2D array^6^. Yet, the exerted electron irradiation^7^ is the main limiting factor to investigating radiation sensitive materials such as oxides^8^, organic hybrids materials^9^ or biological specimens^10^. A further disadvantage of STEM is the relatively long image acquisition time (seconds), under typical pixel-by-pixel conditions, preventing fast in-situ observations, and the hindering of quantitative analysis as drift and scan distortions artifacts arise^11^.

Different strategies have been implemented to increase scanning speeds and minimize beam damage. The first approach relies on developing new probe scanning systems, of lower inductance, coupled with faster scintillator detectors; this allows for 25 frames per second *fps* acquisitions of 512 × 512 pixels size *pxsz* images with atomic resolution^12^. The second makes use of nonrectangular scans to avoid flyback times, i.e., the extra time required to move the beam from the end of one line scan to the beginning of the next; in this way, spiral scans can be used to acquire 100^2^π *pxsz* [001] SrTiO_3_ images at 20 *fps*^13^. The third approach includes several sparse data collection methods which require subsequent reconstruction by inpainting algorithms^14^; their effective acquisition time is directly related to the amount of pixels acquired.Various methodologies produce the required random pixel-sampling required for sparse data image reconstruction, and although all of them can help reducing dose, some offer no benefits on the overall speed. In the case of experiments on block-scanning strategies, time is invested into relocating the electron probe, while the overall acquisition could be prone to data overlap and mismatch^15^. Regarding the addition of a fast beam-blanking system, to avoid collecting some pixels, it takes the same time as a standard full frame image^16^. An effective sparse sampling subroutine then needs to be realized by precise control of the scanning coils^17^. However, until now, this approach has included flyback times aimed at reducing distortions, enforcing a fixed lower bound on the overall acquisition speed.

**Figure 1:**
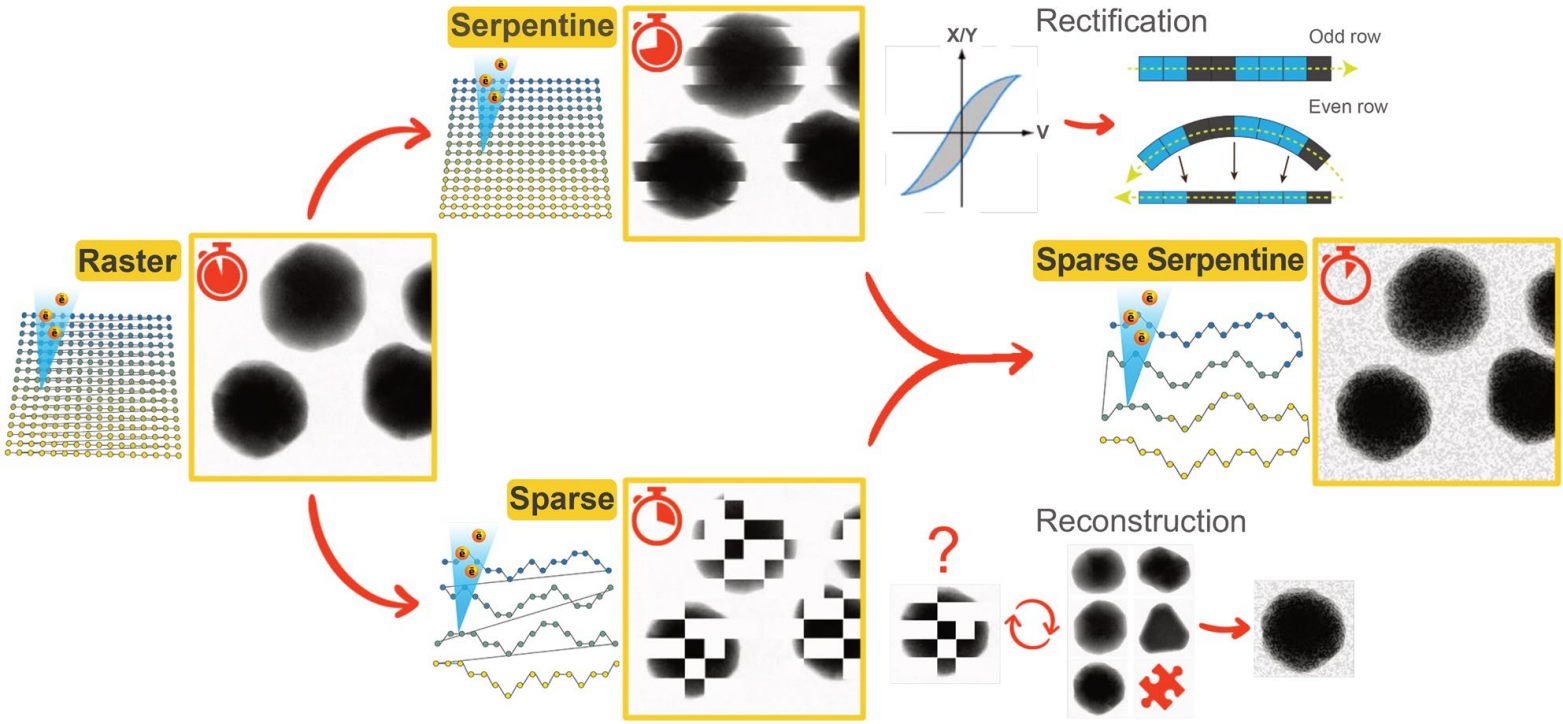
Schematic illustration of different scanning strategies and their relative acquisition time. The standard pixel-by-pixel raster scan of Scanning Transmission Electron Microscopy (STEM) includes the addition of a 'flyback' time to relocate the beam at the beginning of the next row. Via a serpentine scan, the frame rate can be improved by avoiding any dead-time τ, but the rectification of odd and even rows is required to compensate for hysteresis effects of the magnetic scan coils. Sparse imaging results in the recording of fewer pixels through a random-walk scan thus reduce the total time. Here, a reconstruction algorithm is needed to “inpaint” the full frame. Both approaches can be combined to achieve the highest possible STEM image acquisition speed while avoiding an increase in electron dose.

Here, we demonstrated the combination of both sparse and unconventional scanning routines to acquire STEM images at a high spatio-temporal resolution. As depicted in Fig. 1, advantages and disadvantages of serpentine and random walk sub-sampling scanning routines were analyzed separately. Atomic resolution images were chosen for calibration purposes while Au nanoparticles (AuNPs) were used for applicability. In this manner, different experimental parameters and postprocessing strategies could be tested to rectify for scanning distortions and reconstruction artifacts as a function of *T*_*D*_ and *pxsz*. Once these distortions were accounted for, sparse-serpentine 256 × 256 *pxsz* images of AuNPs in solution were achieved at 23 fps. Videos from the different runs are shown in the supplementary information.

## Results and discussions

### Estimation of flyback times

**Figure 2:**
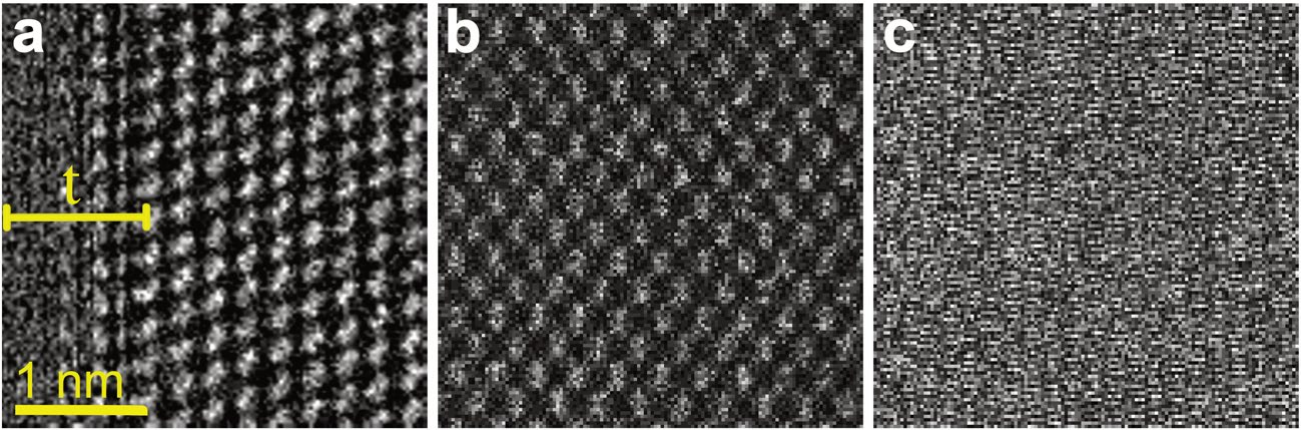
STEM images acquired along a [110] Si using different acquisition routines. (**a**) 128 × 128 raster scan with a dwell time *T*_*D*_ = 10 μs, but no flyback time showing image distortions. (**b**) The addition of τ prevents image artifacts. (**c**) Raw image acquired with a serpentine scan.

The standard scanning pattern implemented for STEM imaging (raster scan) relies on the amplitude recording of the detector signal into a 2D array while the beam moves from left to right and top to bottom. The order is interchangeable, but in essence comprises of a slow and fast axis movement of the electron probe. To avoid distortions and line synchronization issues, after a line scan is finished (trace), the beam is moved immediately towards the beginning of the next line, hence ignoring all signals from the trace back displacement of the beam (retrace). As an example, Fig. 2a shows a 128 × 128 image from [110] Si acquired with a dwell time T_D_ = 10 µs where no extra time has been given for the retrace movement, resulting in an acquisition speed of 6.1 fps. The image distortion can be related to the *T*_*D*_ to find the associated flyback time. In the case of our system, a value of 390 µs was sufficient to avoid artifacts. This dead time τ is in the same range of other microscopes as exemplified in the work of others^18^. Once τ is added into the coordinate files, i.e., repeating the same initial line coordinate over to compensate for the lag, a distortion-free image can be generated (Fig. 2b). As a trade-off, additional electron irradiation is unevenly exerted into the sample as the beam moves back. Moreover, this delay reduces acquisition speed to 4.7 fps. This effect depends on the image dimensions (in pixels) and *T*_*D*_. Thus, for a 1024 × 1024-pixel and a 256 × 256-pixel scan at *T*_*D*_ = 1 µs, the acquisition improvement ratio (non-flyback/flyback) is 1.4 and 2.6 respectively. A way to avoid the flyback time is therefore highly desirable.

The straightforward approach relies on scanning the next line immediately at the end of the current line but moving the beam towards the opposite direction. In that configuration, the trace and retrace signal would correspond to the odd and even rows within a square scan. An example of such a serpentine scan is shown in Fig. 2c, acquired with the same settings as Fig. 2a (6.1 fps). At certain speeds, trace and retrace signals can be completely out of phase, producing a pseudo-noisy image. This distortion arises from ferromagnetic hysteresis effects present in the scanning deflector coils of the microscope. As only the trace signal position is parametrized, the retrace values are assigned to the wrong nominal fast-axis position. The compensation process of odd and even rows would require the use of the former lines as a reference and the analysis of a well-defined periodic sample to rectify the elongation/contraction of the probe pathway.

### Serpentine scan rectification process

**Figure 3:**
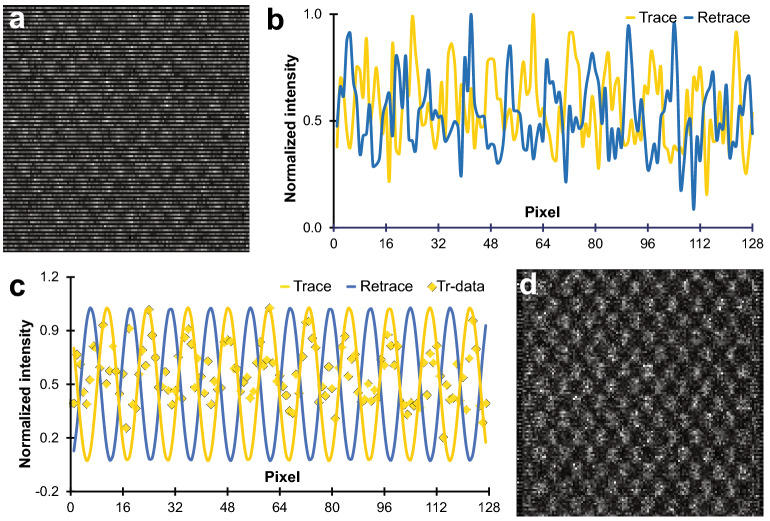
Serpentine scan rectification procedure. (**a**) STEM retrace data (even rows) from Fig. 2c. (**b**) Selected trace and retrace line scans showing maxima mismatch. (**c**) Fourier series fitting of profiles shown in (**b**), trace datapoints are shown for comparison. (**d**) Distortion corrected (rectified) image acquired after minimizing the difference between the trace and retrace signals.

At first glance, the scan presented in Fig. 2c seems worse than the expected raster scan. However, as observed from Fig. 3a (where even rows are displayed separately), it is noticeable that the atomic arrangement between the Si columns is being preserved. A closer inspection, given by the normalized intensity profiles of two consecutive line scans shown in Fig. 3b, highlights the mismatch between maxima, i.e., the column positions. A first approach to correcting for the scanning distortions would be to apply an offset to the retrace values to overlap both intensity profiles. Nevertheless, as demonstrated by the elongation of the atomic distances at higher acquisition frequencies (Fig. S1) and the remnant of artifacts under low *T*_*D*_, a shift is insufficient to correct for the serpentine scan distortions. To find the higher order distortion coefficients, prior knowledge of the Si lattice can be used. Rectification of non-periodical samples is unlikely as curvatures, gaps and features make every line scan unique. Regardless, even images of crystal lattices are prone to artifacts caused by beam damage, drift distortion and emission fluctuations^11^.

In order to avoid noise inhomogeneities and obtain sub-pixel resolution, the trace and retrace signals were represented as a one-harmonic Fourier series. As depicted in Fig. 3c, the fitting process allowed the visualization of the periodic lattice and normalized the intensity and spread of the data. If the trace signal is used as a reference signal, i.e., the nominal fast axis position vector, both trace and retrace intensity vector *y* can be expressed as:

1 $$y_{t} = {\text{a}}_{t0} + a_{t1} \cos \left( {w_{t} x_{t} } \right) + b_{t1} \sin \left( {w_{t} x_{t} } \right)$$

2 $$y_{r} = {\text{a}}_{r0} + a_{r1} \cos \left( {w_{r} \left( {z_{1} x_{t} + z_{2} } \right)x_{t} } \right) + b_{r1} \sin \left( {w_{r} \left( {z_{1} x_{t} + z_{2} } \right)x_{t} } \right)$$

where a_0_, a_1_ and b_1_ model the intercept and amplitude-based Fourier coefficients in the data, and the subscripts *t* and *r* are associated with the trace and retrace signals respectively. The transformation $x_{r} = z_{1} x_{t}^{2} + z_{2} x_{t}$, with rectification coefficients z_1_ (proportional) and z_2_ (offset), subordinates the retrace fast axis to the parametrized $x_{t}$ to correct for compression effects. Once the minimization problem for the intensity vectors is solved, the rectification coefficients can be applied to obtain a rectified image such as in Fig. 3d, where the scan distortions have been corrected. However, some pixels at the edge of each line have been removed. The precise number of trimmed pixels depends mostly on *T*_*D*_.

### Analysis of rectified STEM images

**Figure 4:**
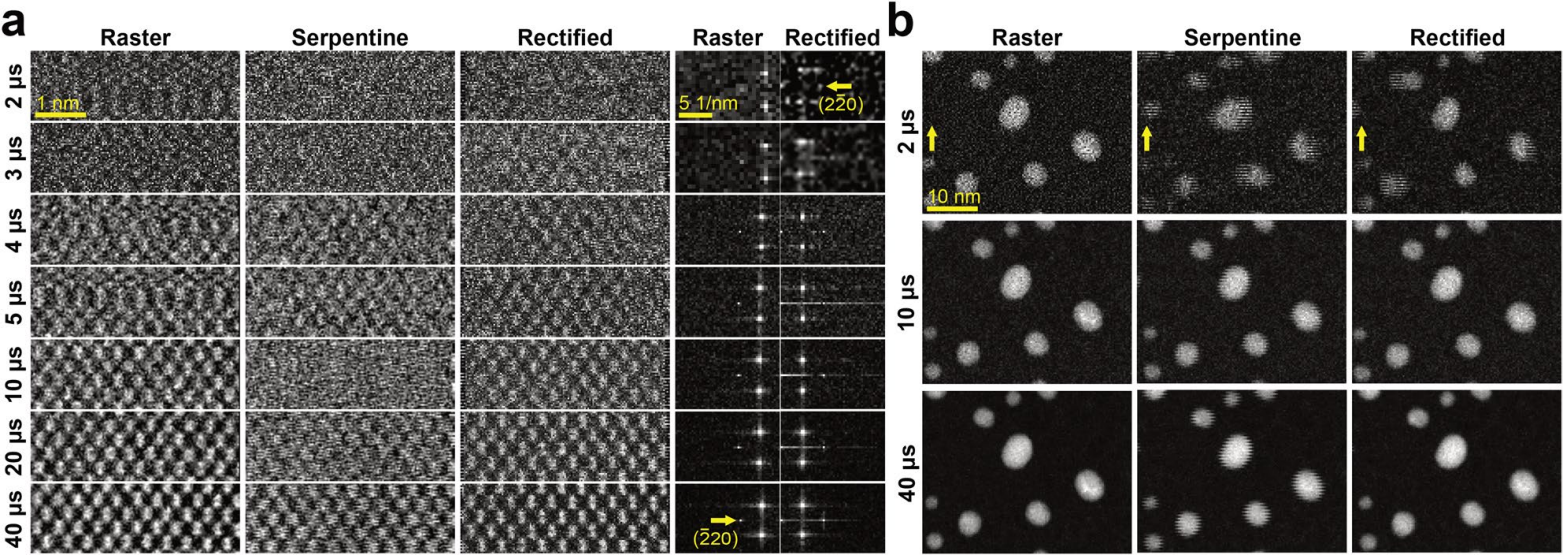
Comparison between rectified and experimental STEM images. (**a**) Atomic resolution images of [110] Si at different *T*_*D*_. The raster and serpentine scans are acquired with τ = 400 and 10 μs, respectively. The Fourier transform of the raster and rectified images highlight the presence of the (220) diffraction spot at 0.192 nm. (**b**) Rectification of STEM images from gold nanoparticles AuNPs using the scan distortions parameterized from (**a**).

The rectification process was tested for both atomic resolution, and high contrast STEM samples. On Fig. 4a a set of 128 × 128 sections compare the effects of *T*_*D*_. As the main scan distortion was produced along the fast axis, the proper location of the (220) planes in the Fourier transform were used to validate the rectification process. Additional runs, where an extra τ = 10 μs was added at the end of each line scan, were also collected to examine the effect of beam acceleration. The values for z_1_ and z_2_ are given in Table S1 and also serve as a measurement of the deviation of the retrace scanning from the nominal scan. As expected by visual inspection of Fig. 4a, as *T*_*D*_ decreased, a larger offset was required to compensate for scan artifacts, until z_2_ reached a maximum at 10 μs. Interestingly, there was not a linear increase of the coefficient values at faster speeds. The coefficients either remain almost the same as for the case of 128-pixel scans or decreased to start ramping up as with other scan sizes. The extrapolation of these parameters into the imaging of high contrast gold nanoparticles (AuNPs) in Fig. 4b confirms this trend; it also verifies that the success in rectification is not related to a local minimum caused by the periodicity of the Si (Fig. 4a). The effect of an added τ, intended to reduce inductance effects from the scanning coils, showed no benefit to the analyzed parameters. For larger image size, scanning distortions became less prominent (Fig. S2); but at the same time, the benefits of using this scanning scheme diminished.

The performance of the serpentine scans is observed in Fig. 4b, where some fluctuations around the AuNPs edges can be detected, for most cases the resulting STEM images do not display noticeable linear distortions. However, at higher frame rates (30.5 fps − *T*_*D*_ = 2 μs) another type of artifact arose, the retrace line started collecting signals from outside the original field of view. As highlighted in Fig. 4b the serpentine scan was imaging an AuNP which was not in the range of the raster scan. The simple axis transformation of the retrace axis was insufficient to completely flatten the extra information from the far edge without affecting the signal distribution of the overall image. The main problem with the serpentine scan is that even with the use of subpixel rectification coefficients, the intensity values need to fit into discrete pixels (rounding). Therefore, increasing the pixel density (size) of the image can help to mitigate these effects, as voltage steps from the scanning coils become less abrupt. This is evident from the 22 mV/px signal adjustments required for a 128-px wide line-scan compared with the 3 mV/px of its 1024-px counterpart.

### Sparse scan reconstruction process

An evident approach to decrease acquisition times is by “simply” recording fewer image pixels. This approach has the advantage that the electron dose is reduced accordingly. As shown in the work of Kovarik et al., in order to overcome scanning distortions during the sparse acquisition, a random vertical perturbation can be added to benefit from the regular movement of the fast axis^17^. Thus, adjusting the scan line-width directly relates to the sparsity level in integer fractions. Once the subsampled images were acquired, the reconstructions were performed using a beta-process factor analysis via expectation maximization (BPFA-EM) algorithm^14,19^. BPFA-EM performs both the dictionary learning and sparse coding steps, and the dictionary is learned from the subsampled input data, this is called ‘blind inpainting’. BPFA is a fully Bayesian conjugate prior, this permits a dataset to be separated into a linear combination of a sparse set of vectors which aims to represent the dataset it was built from. The addition of an expectation maximization algorithm increases the efficiency and reliability of BPFA^20,21^. The resultant reconstruction quality is subject to many variables, such as the sampling percentage, image feature size, selection of reconstruction parameters, *T*_*D*_ and beam current for a constant dose.

### Analysis of reconstructed STEM images

**Figure 5:**
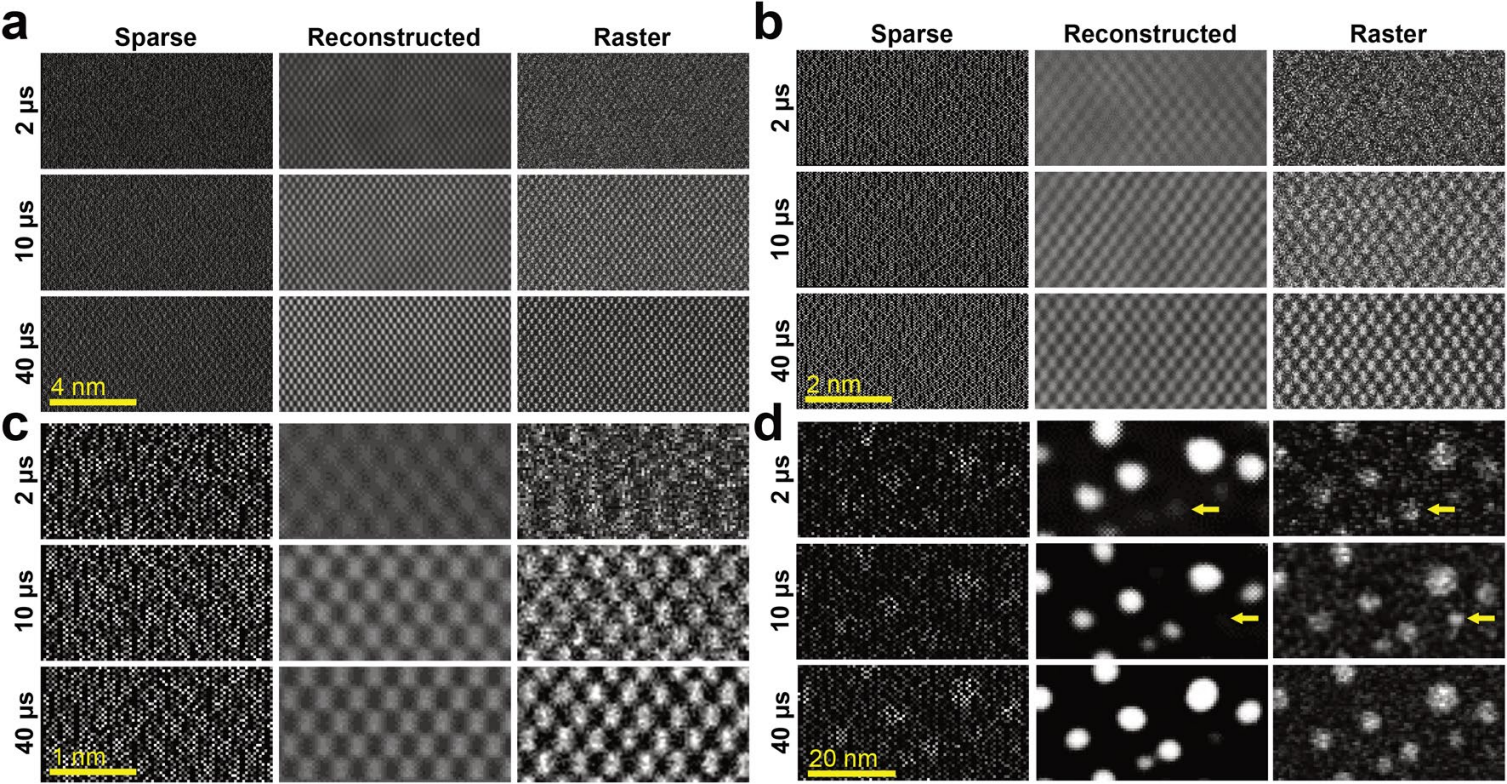
Comparison between reconstructed and experimental STEM images. Atomic resolution images of [110] Si with τ = 400 μs but different *T*_*D*_. The random-walk routine acquires 1/3 of all possible (**a**) 512 × 512, (**b**) 256 × 256 and (**c**) 128 × 128 pixels. The pixel size *pxsz* = 30.1 pm was kept constant. (**d**) Extract from a 128 × 128 scan routine (*pxsz* = 0.77 nm) of the AuNPs at different noise levels in order to promote the appearance of artifacts.

The quality of the reconstructed images was tested for images of 512-pixel, 256-pixel, and 128-pixel width (Fig. 5). The pixel size was kept constant, meaning that the beam movement across the 2D array of coordinates performed the same discrete steps. However, due to the magnification effect of the objective lens, the voltage exerted by the scanning coils differed from 11 to 0.07 mV/μs when reaching scanning speeds of 16.1 and 0.3 fps, respectively. Even as subsampling levels of 3% has been used for inpainting experiments^22^, all sparse images shown here were acquired with 33% of the available pixels in order to be consistent with later AuNPs and in-situ observations. From Fig. 5a–c, the full lattice was easily recovered. As the data acquisition was based on a random walk acquisition scheme, data redundancy can be achieved quickly. The randomness is essential so that specific features can be learned from other areas of the image with better sampling. The algorithm can then transfer this knowledge to poorly filled regions and complete the inpainting process^14^. For that reason, the images with more pixels (512 × 512) exhibit a higher structural similarity index measure (SSIM)^23^ between the reconstruction and their corresponding raster image acquired with *T*_*D*_ = 40 μs (Table S2).

In Fig. 5d, the reconstruction process was applied to a sample with less redundancy, spherical AuNPs. The 128 × 128 scans are shown as they represent the most arduous scanning conditions. As highlighted on the figure, while noise became more prominent, some nanoparticles stopped being resolved as too few pixels within the target AuNP were properly detected. The selection of an appropriate pixel size to match the expected sparsity factor and the minimum feature size (resolution) becomes critical to avoid either oversampling or undersampling. Moreover, the inpainting processing technique can introduce gaussian blurring, reducing the overall sharpness (resolution) of the reconstruction.

### Analysis of rectified reconstructed STEM images

**Figure 6:**
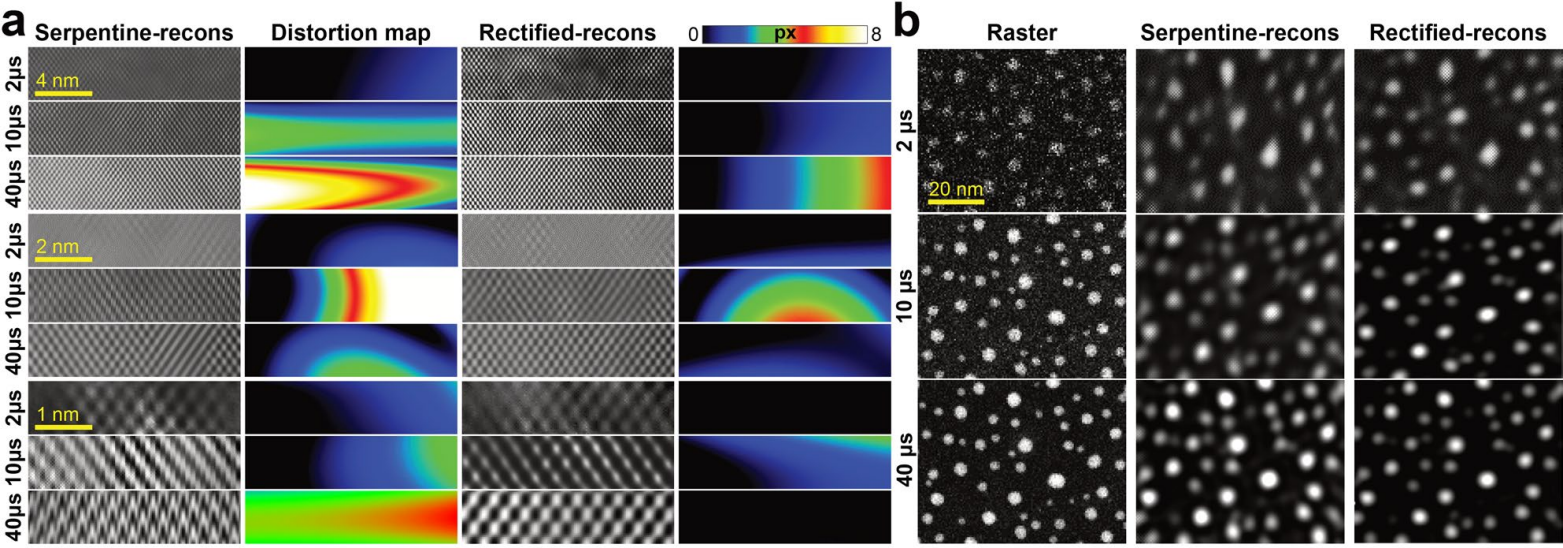
Comparison between rectified-reconstructed and experimental STEM images. (**a**) Difference between Serpentine-reconstructions and Rectified-reconstructions of experimental sparse(1/3)-serpentine [110] Si images at different image size and *T*_*D*_. The color maps quantify the degree of misalignment (in pixels) between the postprocessed atom positions and the ideal lattice. (**b**) Comparison between raster, serpentine-reconstructions, and rectified-reconstructions of the AuNPs.

Both rectified and reconstructed approaches produced STEM images of a quality comparable with that of the raster scan. The combination of both scanning schemes can be used to achieve higher temporal resolution without modifying any of the microscope’s instrumentation. Table S3 shows the achievable *fps* for each STEM strategy and acquisition conditions applied here; using higher sparsity conditions could even bring 256 × 256 scans into video-rate image acquisition speeds (> 24 fps). Figure 6 serves as a way to visualize the distortions produced by the serpentine scan. In Fig. 6a, the acquired sparse(1/3)-serpentine images were reconstructed with and without the rectification process. On smaller scale images, which in turn were acquired faster (92.5, 18.5 and 4.6 fps), scanning distortions can be discerned visually either by the loss of discrete atomic positions (presence of lattice fringes) or by the existence of artifacts altering the atom shape. For 512 × 512, such distortions were negligible, but the atomic rows appear bent due to the hysteresis effects into the retrace scan. In order to visualize the degree of misalignment between non-rectified and rectified reconstructions, a colormap distortion magnitude image quantifies how the distortion (in pixels) varied across the experimental image from the ideal lattice positions^24^. Since the *pxsz* was kept constant, the color scale applies for all images; black values were close to zero while white regions represented large distortions of at least 8 pixels. The increased non-uniformity at larger *T*_*D*_, located at the opposite corner from the initial starting scan position, related mostly to drift instabilities cause by vacuum, thermal, and mechanical fluctuations.

The implementation of the rectification coefficients, obtained in Table S1, improved the fidelity and minimized scan distortions of the reconstructed images as shown by their corresponding distortion colormaps. In Fig. 6b, the same inspection was performed on the AuNPs, the rectification process reduced the elongation over the fast scan axis and preserved the objects’ proper positions and morphology. The *z*_*1*_ and *z*_*2*_ axis transformation remained at different *pxsz*, as the same voltage was exerted by the scanning coils (± 1.45 V) to produce the horizontal and vertical movements of the probe, while the observed magnification was given by the objective lens.

The benefit of these scanning schemes has thus far aimed to reduce acquisition times. Nonetheless, it is important to point out that these methods can be applied to low dose settings when compared with the raster scan approach. Due to the reduced times achieved, sensitive materials, prone to damage by not only the total electron dose but also the dose rate, could be imagined, while also reducing drift and charging effects as the electron irradiation becomes more spread out^25^.

### Demonstration of fast scans on kinetic processes in liquid

**Figure 7:**
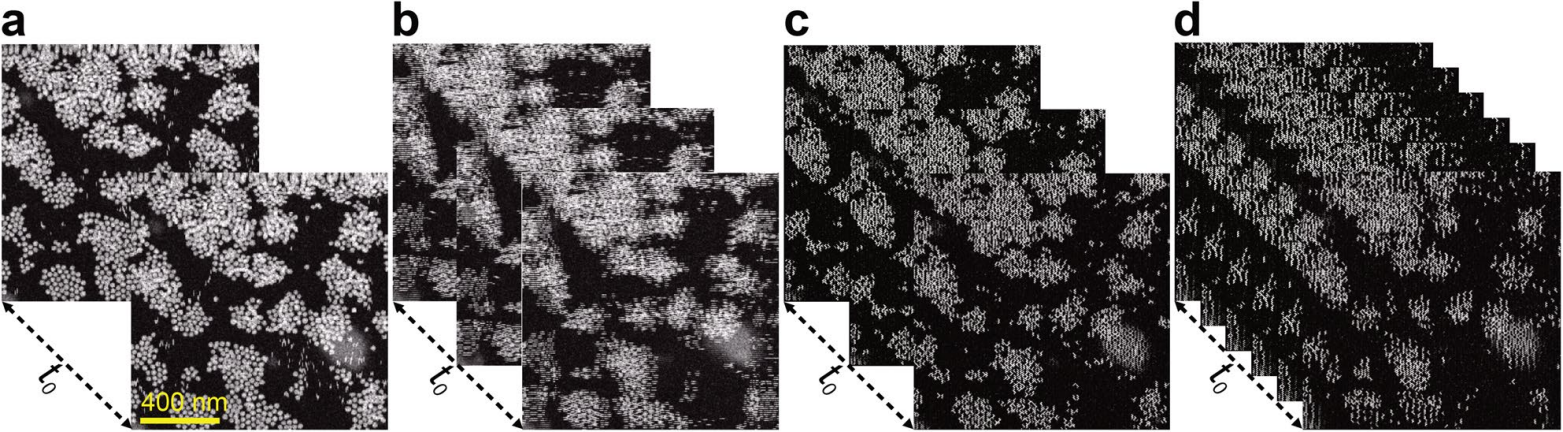
Schematic of sequential image acquisition for in-situ AuNPs in liquid phase TEM. Individual frames acquired from the beam assembly process of colloidal AuNPs in a liquid layer of 700 nm thickness. (**a**) At the same time *t*_*0*_ = 23 ms it takes to acquire two sequential 256 × 256 raster scan images (τ = 400 μs and *T*_*D*_ = 2 μs); the framerate can be improved 1.8, 1.6 and 5.4 times by implementing: (**b**) serpentine, (**c**) sparse 1/3 (τ = 400 μs) and (**d**) sparse(1/3)-serpentine scanning pathways respectively. Further analysis requires the rectification and reconstruction procedures implemented earlier.

In order to apply the selected scanning algorithms to a more dynamic experimental setting, we used AuNPs within a microfluidic chamber assembled in the specimen holder for TEM to observe beam-induced assembly of AuNPs in liquid. The AuNPs were immobilized at the top window (relative to a downward-traveling electron beam) or floated in the liquid and started aggregating with time, forming 2D clusters. Figure 7 show stills from a movie (Movie S1) recorded from the AuNPs in solution. Several AuNPs started to move and aggregated immediately after irradiation with the electron beam. The blurriness observed at the bottom right quadrant of the STEM images was caused by the AuNPs sitting at the lower silicon nitride membrane, the streaks indicate fast-moving nanoparticles, which can only be detected once by the line scan. The movement and formation of one-dimensional chains and the self-assembly of AuNPs has been analyzed extensively^26,27^. The increased framerate has been quantified in Table 1, where the displayed values are related to the standard raster scan. In the case of our 256 × 256 video acquisition with a *T*_*D*_ = 2 μs, the combination of both methods increased the overall speed by 5.4 times with a nominal acquisition time of 4.4 ms. To obtain a full rectified-reconstructed video, each sequential frame needs to be sequentially postprocessed and reassembled^22^. Usage of higher read-out frequency instrumentation or higher sparsity levels could easily push the acquisition time to 1 ms, being limited in practice by sample sensitivity, resolution, and information redundancy. For the latter, the pixel size should ensure that the ratio of the minimum feature size (in pixels) over the sparsity level is greater than one for proper sampling. To adapt these methods to other microscopes, the same calibration procedures employed throughout this work need to be implemented to obtain distortion-corrected fast frames.

**Table 1:** Acquisition improvement ratio between the raster and tested custom scanning strategies as a function of pixel-dwell time *T*_*D*_ and image size in pixels.

	Serpentine					Sparse 1/3					Sparse(1/3)-Serpentine				
*T*_*D*_ (µs)	1	2	5	10	20	1	2	5	10	20	1	2	5	10	20
128 × 128	4.1	2.6	1.6	1.3	1.2	1.2	1.4	1.7	2.0	2.4	12.5	7.8	4.9	4.0	3.5
256 × 256	2.6	1.8	1.3	1.2	1.1	1.4	1.6	2.0	2.4	2.6	7.8	5.4	4.0	3.5	3.3
512 × 512	1.8	1.4	1.2	1.1	1.0	1.6	1.9	2.4	2.6	2.8	5.4	4.2	3.5	3.3	3.1
1024 × 1024	1.4	1.2	1.1	1.0	1.0	1.9	2.3	2.6	2.8	2.9	4.2	3.6	3.3	3.1	3.1

In this work, we demonstrated the feasibility of combining two different electron beam scanning strategies to improve STEM acquisition. The implementation of serpentine scans required thorough calibration of the rectification coefficients in order to assess the behavioral responses of the scanning coils. The application of sub-sampling strategies served as a straightforward approach to reducing electron dose and image acquisition time when sparsity levels avoid oversampling, i.e., when the sample is periodic or the *pxsz* is small enough that redundancy exists. By eliminating flyback times and performing random walk scans, rectified-reconstructed STEM images were acquired at high speeds with a similar quality of standard raster scan images. The methods described herein have a direct application on the fields of low-dose imaging and single-particle tracking experiments in order to obtain reliable information on kinetic processes in liquid.

## Methods

The calibration sample consisted of a Si lamella prepared using a focus ion beam (FIB) and oriented into the [110] zone axis. Further analysis was performed with a gold on carbon resolution test specimen comprised of evaporated AuNPs of 5 ± 1 nm diameter. For application purposes, a liquid flow TEM holder (Poseidon, Protochips Inc., NC, USA) was used to image 50-nm diameter AuNPs in cyclohexane. The thickness of the liquid layer was approximated by the log-ratio of the detected current before and after liquid addition. Images were acquired using a probe-corrected STEM (ARM 200F, JEOL, Japan) equipped with a cold field emission gun (FEG) operated at 200 kV. The high angle annular darkfield (HAADF) detector signal was collected from an electron probe with a 20 mrad convergence semi-angle and a 68–280 mrad collection semi-angle. The beam current was kept at 28 pA for the dry samples and at 66 pA for the liquid experiment. The resulting electron flux was scaled by the *pxsz* yielding 2.0 × 10^9^, 2.6 × 10^7^ or 1.7 × 10^5^ e^−^/Å^2^s for the Si, Au, and liquid samples, respectively. The scanning coils of the microscope were controlled by a DE-FreeScan scan generator (Direct Electron, CA, USA) connected into the JEOL microscope computer. The maximum analog data converter rate of this system is 1 MHz associated with an equivalent dwell time of 2 µs. The scan-files coordinates were created with a custom program (written in Matlab, Mathworks, MA, USA) in order to specify the beam position pathways.

After data acquisition, scan distortions were rectified with a custom Matlab program that selects consecutive trace/retrace line scans from an atomic resolution image and applies a Fourier series fitting process to smooth and normalize the data. The difference between the two intensity vectors (trace/retrace) is minimized, computing a nonlinear regression of the responses produced by applying a perturbation of the position of the retrace vector. The amount of this offset is given as *z*_*1*_ and *z*_*2*_. Once the coefficients are known, they can be used in images acquired at the same conditions. Image reconstructions were performed using the Nuxutra Image-Inpainting software (Sivananthan Laboratories, IL, USA) which applies a BPFA-EM image inpainting algorithm to recover a full pixel size image. Typical parameters for the presented reconstructions were: dictionary columns *k* = 24, patch size length *b* = 24, number of passes *n*_*epoch*_ = 2, sparsity probability *p*_*i0*_ = 2%, number of patches per learning batch *n*_*batch*_ = 256 and number of patches per reconstruction batches *n*_*batch-r*_ = 50,000. The SSIM of a pair of two images (reconstruction and reference), was computed by Fiji ImageJ (Ver.1.53) (NIH) in order to obtain a rubric for quantification of image quality degradation. The distortion colormaps were producing with a Digital Micrograph software (Gatan Ametek, CA, USA) open-source script (University of Wollongong, NSW, Australia) which performs a 2D polynomial fit in order to map the relationship between the ideal lattice and the distorted one. The frame-by-frame acquisition of the AuNPs in liquid was achieved by screen recording the image display window of the native software of the DE-FreeScan scan generator. The recording conditions were 512 × 512 pixels at 60 fps.

## Supplementary Information

Supplementary Information 1.

Supplementary Video 1.

Supplementary Information 2.
