# Supplementary material for: High temporal-resolution scanning transmission electron microscopy using sparse-serpentine scan pathways
Source: Sci Rep. 2021 Nov 22;11:22722. doi: 10.1038/s41598-021-02052-1 (PMC8608981; doi:10.1038/s41598-021-02052-1)
Supplement: Supplementary file 1 — Supplementary Information 1. [file 41598_2021_2052_MOESM1_ESM.docx]

**Captions of supplementary movie S1**

**Movie S1.** Acquisition of in-situ AuNPs in liquid phase scanning transmission electron microscopy. Montage of four screen recordings of the beam-assembled process of AuNPs in liquid. The improvement on acquisition speed for the four scanning pathways investigated follow the trend show in Table 1. The experimental conditions are described in the caption of Figure 7.
