# Supplementary material for: High temporal-resolution scanning transmission electron microscopy using sparse-serpentine scan pathways
Source: Sci Rep. 2021 Nov 22;11:22722. doi: 10.1038/s41598-021-02052-1 (PMC8608981; doi:10.1038/s41598-021-02052-1)
Supplement: Supplementary file 3 — Supplementary Information 2. [file 41598_2021_2052_MOESM3_ESM.pdf]

Supplementary Information

High temporal-resolution scanning transmission electron microscopy using sparse-serpentine scan pathways

Eduardo Ortega, Danny Nicholls, Nigel D. Browning & Niels de Jonge

Contents

Supplementary Tables S1- S3

Supplementary Figures S1, S2

	512 x 512 px				256 x 256 px				128 x 128 px			
T_D	z_1	z_2	z_1	z_2	z_1	z_2	z_1	z_2	z_1	z_2	z_1	z_2
2	-0.004	0.80	-0.005	0.89	0.018	4.15	-0.031	4.05	-0.041	4.02	0.082	4.23
3	-0.001	0.61	-0.001	0.64	-0.008	0.48	0.015	0.39	-0.018	4.24	0.005	4.45
4	-0.003	1.64	-0.002	1.39	-0.001	0.55	-0.004	0.66	-0.075	4.45	-0.020	4.19
5	0.000	0.94	0.000	1.01	0.004	0.04	-0.004	0.05	-0.026	0.32	-0.014	0.50
10	0.001	1.44	0.000	1.50	0.000	4.27	0.002	4.78	0.004	5.88	-0.014	5.67
20	-0.001	2.07	0.000	2.05	0.002	4.03	0.001	4.31	0.001	3.74	-0.011	4.40
40	0.001	1.58	0.000	1.90	0.000	2.47	0.003	2.50	0.003	2.02	0.002	2.39
(μs)	$\tau = 0$		$\tau = 10$		$\tau = 0$		$\tau = 10$		$\tau = 0$		$\tau = 10$	

Table S1. Rectification coefficients z_1 (proportional) and z_2 (offset) between different serpentine scans with or without dead time τ.

	[110] Si			AuNPs		
T_D	2 µs	10 µs	40 µs	2 µs	10 µs	40 µs
128x128	0.115	0.110	0.239	0.645	0.670	0.647
256x256	0.251	0.323	0.260	0.663	0.694	0.717
512x512	0.308	0.530	0.623	-	-	-

Table S2. Structural similarity index measure (SSIM) between the reconstruction and the equivalent raster STEM image acquired with T_D = 40 µs.

	Raster (τ = 490 µs)						
128x128	11.9	10.0	8.6	7.5	4.7	2.6	1.4
256x256	4.3	3.3	2.7	2.3	1.3	0.71	0.37
512x512	1.4	1.0	0.80	0.66	0.35	0.18	0.09
1024x1024	0.40	0.28	0.22	0.18	0.09	0.05	0.02
	Serpentine						
128x128	30.5	20.3	15.3	12.2	6.1	3.1	1.5
256x256	7.6	5.1	3.8	3.1	1.5	0.76	0.38
512x512	1.9	1.3	1.0	0.76	0.38	0.19	0.10
1024x1024	0.48	0.32	0.24	0.19	0.10	0.05	0.02
	Sparse 1/3 (τ = 490 µs)						
128x128	16.1	14.8	13.7	12.8	9.5	6.3	3.7
256x256	6.9	6.0	5.3	4.7	3.1	1.9	1.0
512x512	2.6	2.2	1.8	1.6	0.93	0.52	0.27
1024x1024	0.91	0.69	0.56	0.47	0.26	0.14	0.07
	Sparse Serpentine 1/3						
128x128	92.5	61.7	46.2	37.0	18.5	9.2	4.6
256x256	23.1	15.4	11.6	9.2	4.6	2.3	1.2
512x512	5.8	3.9	2.9	2.3	1.2	0.58	0.29
1024x1024	1.4	1.0	0.72	0.58	0.29	0.14	0.07
T_D (µs)	2	3	4	5	10	20	40

Table S3. Acquisition speed in frames per second (fps) for four scanning routines at different T_D and image dimensions (px).

Supplementary Figures

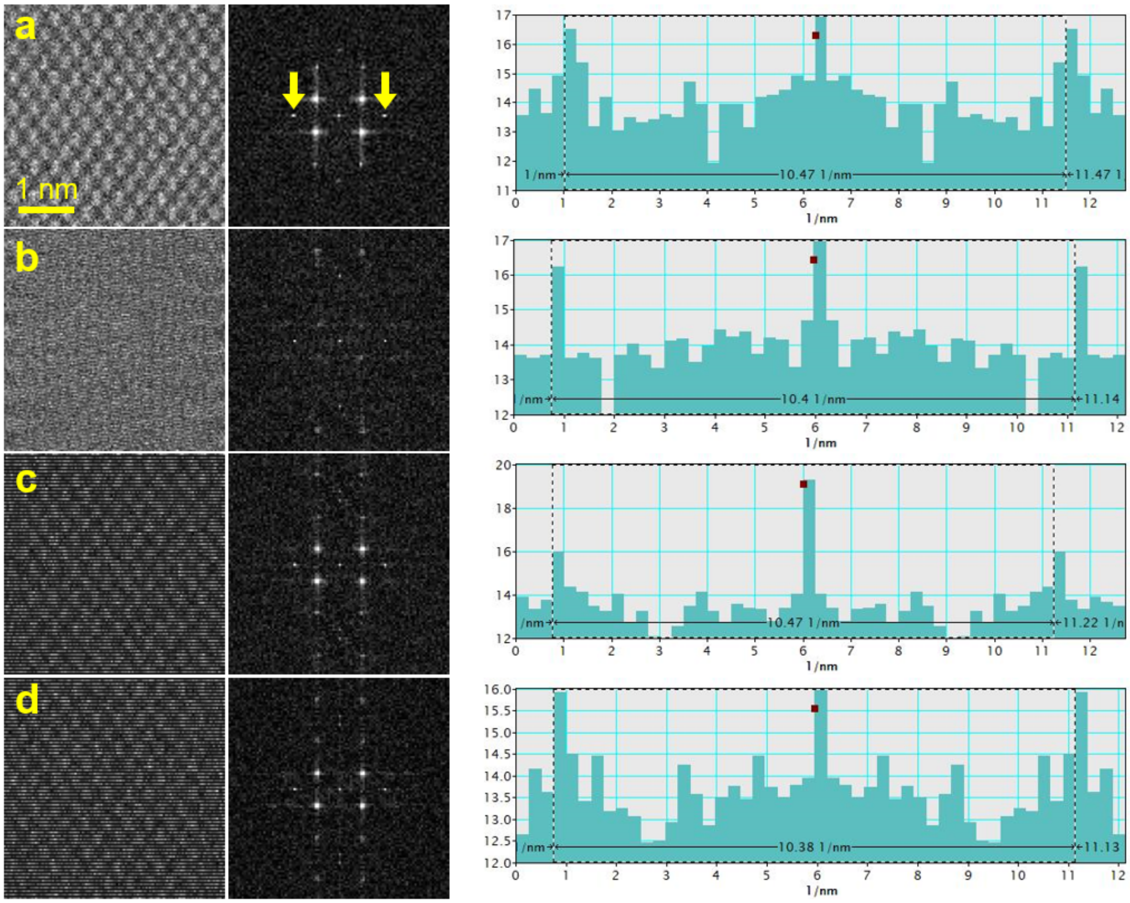

Figure S1. Distortions present on a serpentine scan. 128x128 Atomic resolution images of [110] Si at different T_D = 10 μs. The Fourier transform of the raster and serpentine images highlight the presence of the (220) diffraction spot. The line profile over the x direction reads: (a) 0.191 nm for the raster. (b) 0.192 nm for the serpentine scan, (c) 0.191 nm for the odd rows of (b) and (d) 0.193 nm for the even rows.

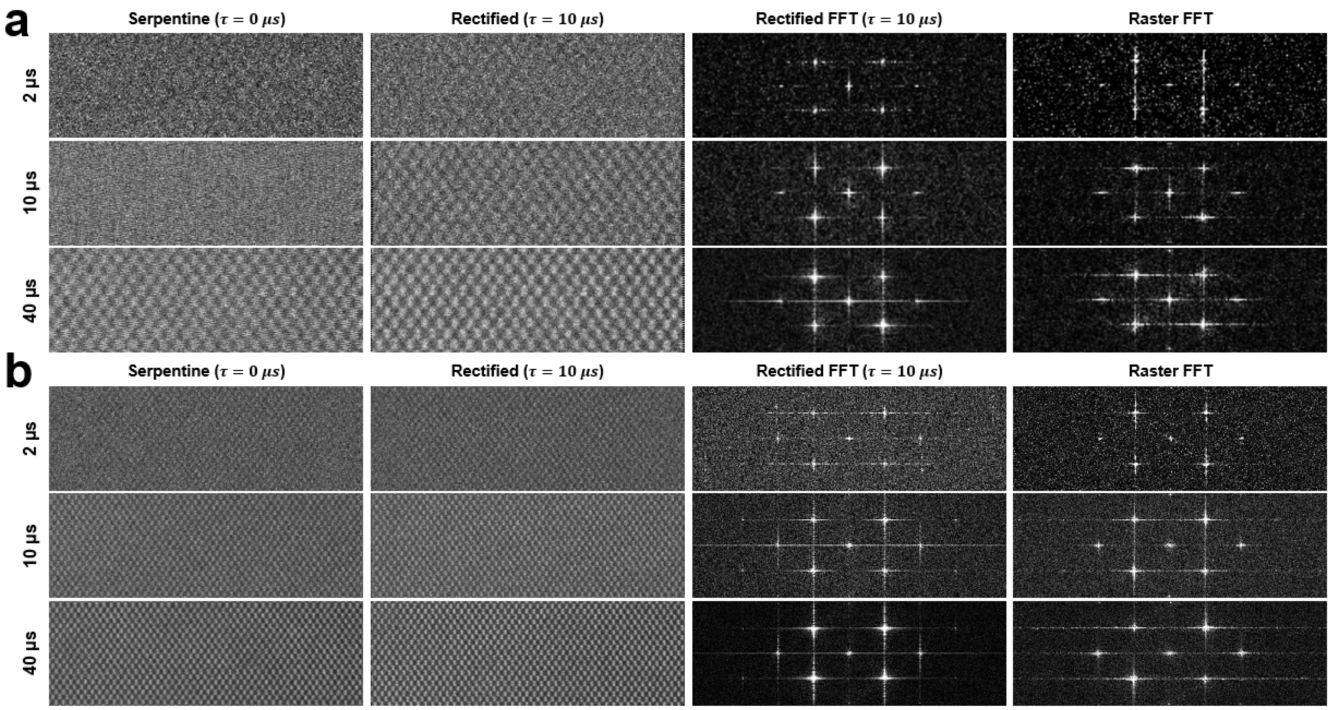

Figure S2. Low distortions experienced by large serpentine scans. Atomic resolution images of [110] Si at different T_D. The Fourier transform of the serpentine and rectified images show no mismatch for the position of the (220) diffraction spot at 0.191 nm. (a) The case for 256x256 images. (b) 512x512 px.
